# Experiences of Community-Living Older Adults Receiving Integrated Care Based on the Chronic Care Model: A Qualitative Study

**DOI:** 10.1371/journal.pone.0137803

**Published:** 2015-10-21

**Authors:** Sophie L. W. Spoorenberg, Klaske Wynia, Andrea S. Fokkens, Karin Slotman, Hubertus P. H. Kremer, Sijmen A. Reijneveld

**Affiliations:** 1 Department of Health Sciences, Community & Occupational Medicine, University Medical Center Groningen, University of Groningen, Groningen, the Netherlands; 2 Department of Applied Research in Care, University Medical Center Groningen, University of Groningen, Groningen, the Netherlands; 3 Department of Neurology, University Medical Center Groningen, University of Groningen, Groningen, the Netherlands; University of Stirling, UNITED KINGDOM

## Abstract

**Background:**

Integrated care models aim to solve the problem of fragmented and poorly coordinated care in current healthcare systems. These models aim to be patient-centered by providing continuous and coordinated care and by considering the needs and preferences of patients. The objective of this study was to evaluate the opinions and experiences of community-living older adults with regard to integrated care and support, along with the extent to which it meets their health and social needs.

**Methods:**

Semi-structured interviews were conducted with 23 older adults receiving integrated care and support through “Embrace,” an integrated care model for community-living older adults that is based on the Chronic Care Model and a population health management model. Embrace is currently fully operational in the northern region of the Netherlands. Data analysis was based on the grounded theory approach.

**Results:**

Responses of participants concerned two focus areas: 1) Experiences with aging, with the themes “Struggling with health,” “Increasing dependency,” “Decreasing social interaction,” “Loss of control,” and “Fears;” and 2) Experiences with Embrace, with the themes “Relationship with the case manager,” “Interactions,” and “Feeling in control, safe, and secure”. The prospect of becoming dependent and losing control was a key concept in the lives of the older adults interviewed. Embrace reinforced the participants’ ability to stay in control, even if they were dependent on others. Furthermore, participants felt safe and secure, in contrast to the fears of increasing dependency within the standard care system.

**Conclusion:**

The results indicate that integrated care and support provided through Embrace met the health and social needs of older adults, who were coping with the consequences of aging.

## Background

Current healthcare systems are insufficiently equipped to meet the broad range of healthcare and social needs of older adults, due to fragmentation of care, poor coordination of care, and lack of patient involvement [1–3]. The greatest limitation faced by current healthcare systems is their disease-oriented approach (which targets care services at separate health problems), combined with their focus on delivering healthcare to people with acute and short-term diseases [3, 4]. At present, however, more than 50% of those 75 years of age and older suffer from multiple chronic diseases [5, 6]. The majority of these patients rely on several healthcare professionals [7, 8], as they require assistance in various domains [9]. This fragmented care may have negative consequences, including misunderstanding by the patient, adverse drug events, impaired treatment participation, and even treatment errors [7, 9]. It is therefore essential to transform the provision of healthcare to the older population into a coherent system focused on community-based long-term care [10]. Such care should consider all health-related aspects, and it should be tailored to the specific situations of individual patients [6, 11, 12].

“Integrated care” models might offer a solution to the fragmentation in the healthcare system. These models are purported to be patient-centered by providing continuous and coordinated care and by taking into account the needs, preferences, and environment of each patient [13, 14]. One commonly used model of integrated care is the Chronic Care Model (CCM) [15], as acknowledged by the World Health Organization [11]. The CCM aims to improve the quality of care and patient outcomes by providing proactive, patient-centered, and integrated care. It links community services to the healthcare system and describes four interdependent key elements: self-management support, delivery system design, decision support, and clinical information systems. Implementing these elements is necessary for achieving productive interactions between an informed and activated patient and a prepared, proactive team of professionals. These productive interactions can then lead to better outcomes [16].

The investigation of whether integrated care models are truly patient-centered calls for both quantitative and qualitative approaches [17]. To date, mainly quantitative studies have been conducted to evaluate the relative patient-centeredness of care, as assessed from the patient perspective [18–20]. Qualitative research would provide greater detail on the personal experiences of patients. These experiences could provide detailed insight into the impact of integrated care models and the extent to which the model fulfils the needs of patients with regard to aging-related health problems, and they could suggest ways to improve the model [21]. At present, qualitative studies on integrated care mainly focused on the experiences of professionals (e.g. [22]), project leaders (e.g. [23]), or specific patient populations (e.g. [24]). One study that explored the experiences of chronically ill patients with integrated care showed that patients appreciated “the coordination within and across teams and with community resources, continuity and sharing of information, and patient engagement” [25]. Experiences of older adults with integrated care, however, are limited and solely focus on specific elements of care, such as home visits [26], or involvement in care [27]. Only one qualitative study was found among older patients and patients with diabetes which showed that person-centeredness–viewed as “being acknowledged, respected, understood, seen, and heard”–is an essential element of integrated care [28]. Qualitative studies exploring the opinions and experiences of older adults with regard to CCM-based integrated care are lacking.

The aim of this study was therefore to evaluate the opinions and experiences of community-living older adults with regard to CCM-based integrated care and support, in addition to determining the extent to which such services meet their needs. The study focused on the following research questions: 1) How do older adults experience the effects of aging? and 2) How do older adults experience the care and support offered by a CCM-based integrated care model?

### Integrated care model: Embrace

Embrace (in Dutch: *SamenOud* [aging together]) is a recently developed population-based integrated care model for community-living older adults [29]. It combines the CCM with a classification of care needs, based on the risk profiles developed by the Kaiser Permanente (KP) Triangle, a population health management model [30]. Embrace aims to provide comprehensive, patient-centered, proactive, and preventive care, in addition to supporting all adults 75 years of age and older within the context of community care. Its ultimate goal is to prolong the ability of older adults to age in place by meeting their needs by supporting self-management, detecting changes in health status at an early stage, and preventing the escalation of health-related problems.

Older adults can be classified into three risk profiles, based on the KP Triangle, as determined by annual screening with self-report questionnaires. The profile “Robust” includes adults without complex care needs and with a relatively low frailty level. The profile “Frail” includes those with a higher level of frailty and who are at risk of developing complex care needs. The profile “Complex care needs” includes older adults with complex care needs. A multidisciplinary Elderly Care Team–consisting of a general practitioner, an elderly care physician, and two case managers (district nurse and social worker)–provides individualized, proactive, and preventive care and support to the older adults.

Robust people are invited to follow a self-management support and prevention program focusing on staying healthy and independent for as long as possible. The program includes regular Embrace community meetings, in which self-management abilities are encouraged and during which local healthcare and welfare organizations provide information on health maintenance, physical and social activities, and dietary recommendations. Robust people are encouraged to contact the Elderly Care Team in case of changes in their health or living situations.

In contrast, frail people and those with complex care needs receive individual support from a case manager, and they are encouraged to follow the self-management support and prevention program. Case managers frequently visit these people at home, assessing their situations, developing individual care and support plans in cooperation with individual clients, implementing these plans, monitoring changes in their medical, psychosocial, and living situations, and navigating the realization of these plans. During monthly meetings, the Elderly Care Team discusses and evaluates the health status and social situations of the older clients. If necessary, proactive steps are taken, in dialogue with the client, to prevent deterioration.

The Embrace model was introduced in community-based elderly care and examined in a randomized controlled trial with an intervention period of twelve months, starting in January 2012. To this end, fifteen Elderly Care Teams from three municipalities in the province of Groningen (in the north of the Netherlands) were trained in working according to the model, and 755 community-living older adults received integrated care and support according to the Embrace model.

## Methods

### Study design

A qualitative study based on the grounded theory approach [31] was conducted. Data were collected by trained interviewers (ASF, KS) through semi-structured interviews [32] conducted eight to ten months after the participants had started receiving Embrace care and support. The methods were defined according to the Consolidated Criteria for Reporting Qualitative Research (COREQ) checklist [33] (S1 Table).

### Study sample

Older adults who were assigned to the intervention group in the Embrace randomized controlled trial were eligible for inclusion in this qualitative study. To obtain a diverse study sample that represented a broad range of experiences, maximum-variation sampling [34] was applied, taking into account the participant’s gender, the Embrace risk profile, and the degree of urbanization of the municipality in which the participant was living. At least eighteen participants had to be included to cover all possible combinations of these three characteristics. Eligible older adults had to be capable of reflecting on their experiences with Embrace. They were invited to participate regardless of their satisfaction with Embrace, if known in advance. Frail older adults and those with complex care needs were invited to participate in the study by their case managers. Robust participants were recruited face-to-face by Embrace project managers during Embrace community meetings. Eligible participants were recruited consecutively. Researchers evaluated the range of participant characteristics after the first six interviews and specified two additional criteria for inclusion: living situation (with or without partner) and educational level.

All of the individuals who were invited agreed to participate in the interviews. Prior to the start of the interview, the interviewers provided the participants with information on the aims of the interview, emphasizing the participant’s right to stop the interview at any time and guaranteeing anonymity. If a participant’s partner was also participating in Embrace, a double interview was conducted.

The method yielded a diverse study sample in terms of the selection criteria (Table 1). In all, 23 community-living participants between the ages of 75 and 89 were interviewed during eighteen interviews, five of which were double interviews with couples. Eight participants were living alone, five were living with others, and five were living with partners who were also participating in Embrace. In the latter cases, both partners participated in the interviews. The double interviews included two couples in which both spouses were robust, two couples of which both had complex care needs, and one couple in which one spouse had complex care needs and the other was classified as robust. The educational level of most participants was medium (57%) or low (39%), with only one highly educated (4%) male participant.

**Table 1:** Characteristics of participants (n = 23).

Interview number^1^	Gender	Age	Embrace profile	Living situation	Degree of urbanization	Educational level^2^
1	Female	86	Complex care needs	With partner	Urbanized rural	Low
1	Male	88	Complex care needs	With partner	Urbanized rural	Medium
2	Male	88	Complex care needs	With partner	Urbanized rural	High
3	Male	87	Frail	Single	Industrial	Low
4	Female	76	Frail	With partner	Industrial	Low
5	Male	86	Complex care needs	Single	Rural	Low
6	Male	85	Complex care needs	With partner	Industrial	Medium
6	Female	85	Complex care needs	With partner	Industrial	Low
7	Female	86	Complex care needs	Single	Industrial	Medium
8	Female	80	Frail	Single	Rural	Medium
9	Female	87	Frail	With partner	Urbanized rural	Low
10	Female	88	Robust	Single	Rural	Medium
11	Male	85	Robust	With partner	Urbanized rural	Low
11	Female	89	Complex care needs	With partner	Urbanized rural	Low
12	Male	81	Frail	Single	Urbanized rural	Medium
13^3^	Male	76	Robust	With partner	Urbanized rural	Medium
14	Female	75	Robust	With partner	Urbanized rural	Medium
14	Male	77	Robust	With partner	Urbanized rural	Medium
15	Female	78	Complex care needs	Single	Rural	Medium
16	Male	85	Robust	With partner	Industrial	Medium
16	Female	83	Robust	With partner	Industrial	Medium
17	Female	81	Frail	Single	Rural	Low
18	Female	78	Robust	With partner	Industrial	Medium

^1^Interview numbers were assigned according to the date of the interview.

^2^Low = Primary school (or less) or lower vocational training; Medium = Secondary school/vocational training; High = Higher vocational education or university.

^3^The partner was present during the interview.

### Data collection

Baseline RCT measurements (performed between October and December 2011) provide data on the background characteristics of participants. The participants’ Embrace profiles and living situations were verified again right before the inclusion of participants.

Interviews were conducted following a semi-structured interview guide, which was developed jointly by experts in qualitative research (GJD, NMHK, SFM, AV) and the Embrace researchers (ASF, KS, SLWS, KW). An explorative interview with one of the Embrace case managers (SO; district nurse) provided additional input for the guide. After the interview guide was finalized during a consensus meeting with the research group (ASF, KS, SLWS, KW), it was tested in two pilot interviews with Embrace participants. Only minor adjustments were made.

Interviews addressed the participants’ experiences with the effects of aging using questions about health, well-being, living situations, daily activities, and the healthcare services they had received. The participants’ experiences of receiving care and support by Embrace were then explored through questions about the Embrace professionals, about the benefits of participating, and about their opinions regarding what would constitute an ideal healthcare situation. Interviewers used open-ended questions with follow-up questions to gain detailed insight, and they kept track of the issues discussed. Interviews were performed during home visits; they were audio-recorded and lasted 60 to 90 minutes. No other individuals were present during the interviews, except for one spouse who was not participating in Embrace (Interview 13; Table 1).

### Data analysis and reporting

**Fig 1:**
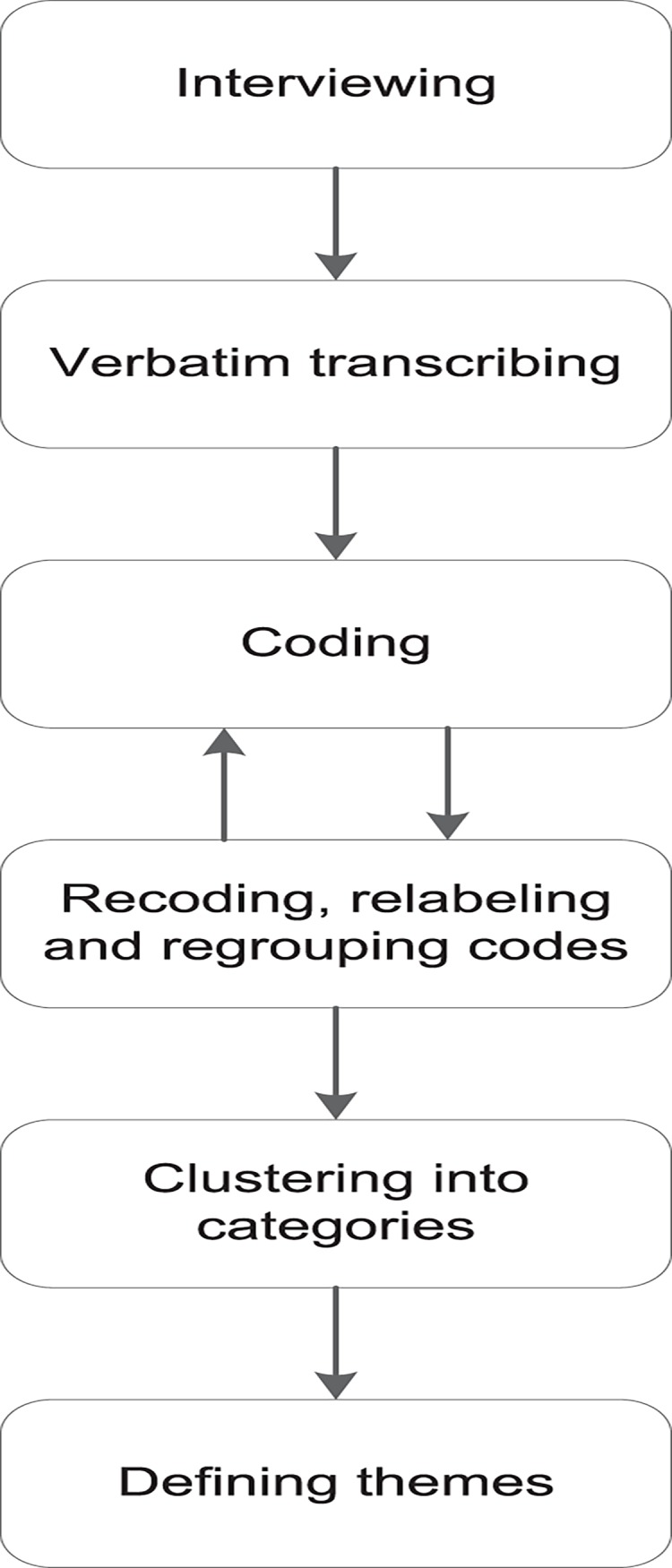
Flowchart of the data analysis and reporting procedure.

Fig 1 presents the flowchart of the data analysis and reporting procedure. All recorded interviews were transcribed verbatim by a research assistant (RB). The transcripts of all interviews were reviewed for completeness and accuracy by the interviewer involved, and they were revised if necessary. Both interviewers read three randomly selected interview transcriptions in order to obtain a sense of the whole.

An initial code book was developed by the interviewers based on the topic guide and the coding of the two pilot interviews. Each code was immediately defined in a short note, and definitions were modified during the coding process. Multiple coding was applied [35]: the two pilot interviews were coded independently by the interviewers. To minimize subjectivity, both interviewers and the first author (SLWS) checked parts of these coded interviews. All other interviews were coded by the interviewer involved, and newly emerged codes were added to the code book. Data saturation was reached after eighteen interviews, as no new codes emerged, and the eighteen interviews provided sufficient information to answer the research questions [36].

After coding all interviews, the accuracy of the codes in relation to the interview quotes was checked by the interviewers (ASF, KS). If necessary, codes were recoded, relabeled, or regrouped under other codes. The final codes were then imported into Excel spreadsheets, including summaries of labeled text fragments and illustrative quotes. Code frequencies were determined, and codes were clustered into categories. Subsequently, themes were deduced from the data and discussed by the researchers (KS, SLWS, KW) until consensus was reached. The main findings for each theme were collected, and special attention was paid to the possible differences in experiences between participants from the three Embrace profiles and between participants who were living alone and those who were living with a partner.

Illustrative quotes from the interviews were selected for presentation in this article, accompanying interview excerpts are provided in the S1 File. The code consists of the first letter of the participant’s profile (R = robust; F = frail; C = complex care needs), the interview number (in order of date), and a second letter representing gender (F = female; M = male), to distinguish between spouses in case of a double interview. For example, code *R18F* refers to a robust, female person who participated in the eighteenth interview (Table 1). Text within the quotes enclosed in square brackets was inserted by the authors in the interest of comprehensibility.

The transcribed interviews were analyzed using Kwalitan 6.0 software. Because the pilot interviews were of sufficient quality, they were also included in the final analysis.

### Ethics statement

The Medical Ethical Committee of the University Medical Center Groningen has assessed the study proposal of the Embrace randomized controlled trial and concluded that approval was not required (Reference METc2011.108). All participants provided written informed consent.

## Results

Responses regarded two separate focus areas (Table 2): experiences with aging and experiences with Embrace.

**Table 2:** Focus areas, themes, and subthemes.

Experiences with aging	Experiences with Embrace
Struggling with health	Relationship with the case manager
Increasing dependency	Equality
Dependency on assistive devices	Confidentiality
Dependency on informal care	Interactions
Dependency on professionals	Being supported
Independent living	Being monitored
Decreasing social interaction	Being informed
Loss of control	Being encouraged
Fears	Feeling in control, safe, and secure

### Experiences with aging

We found clear differences between the participants from the three Embrace profiles with regard to their experiences with the consequences of aging. Robust participants felt in general healthy, but they feared the consequences of aging (e.g. progressive deterioration in health, increasing dependency, and loss of control). In contrast, frail participants and those with complex care needs seemed to struggle with the consequences of aging, including deteriorating health, increasing dependency, decreasing social interaction, and loss of control.

#### Struggling with health

Individuals from the three profiles differed widely in their descriptions of their health. The robust participants were positive about their health, even if they were experiencing physical or mental symptoms. These symptoms did not affect their daily functioning, nor did they play an important role in their lives. The greatest wish of the robust participants was to stay healthy without impairments. In contrast, the frail participants and those who had complex care needs were confronted with deteriorating health, describing their health as “not so good” or “poor.” *“But to say ‘I feel fit*,*’ no*, *I won’t ever be able to say that again*.*” (C15F)*

Participants were ambivalent in accepting their health status. Most had resigned themselves to their physical and mental deterioration, stating that they had accepted their current health status and avoided thinking about future deterioration. *“No*, *what will be*, *will be*. *[…] Luckily*, *we don’t know what the future will bring*.*” (F3M)* Nevertheless, many participants (particularly those with complex care needs) expressed a desire for “better health.” In addition, participants from all three profiles described specific fears related to their deteriorating health (e.g., continuous fear following a life-threatening condition or fear of a stroke). Most of the participants also indicated that they feared falling due to loss of mobility, and that they had become more cautious when moving. *“I feel… I’m generally more anxious*, *particularly in the dark and when driving*, *that sort of thing*.*” (F9F)*

Participants rarely mentioned death during the interviews. When it was discussed, however, the participants differed in their attitudes toward death. Some of the participants with multi-morbidity spoke of death as a merciful release. Another participant with complex care needs said that he did not yet want to die, although he did think about the end of his life.

#### Increasing dependency

Dependency was a major recurring topic. All participants expressed the wish to stay independent for as long as possible and to continue doing as much as possible without the help of others. Those who were not yet dependent on others expressed the fear of future dependency. *“You become so dependent if you require assistance with everything*.*” (C15F)* Differences emerged between the participants in the three profiles with regard to dependency levels. Most of the robust participants were still managing their daily lives without any help from others, although some feared becoming a burden to others. *“That you’re not dependent on someone else […] because you see it here from close up*: *someone arrives in the morning to wash you*, *at lunch time to make you a hot meal and wash up again*, *and then in the evening to get you ready for bed*. *I hate the idea of that*.*” (R10F)* In contrast, most of the participants with complex care needs and some frail participants were dependent on others because of decreasing mobility or impaired cognitive functioning. Some found it difficult to accept the fact that they could not function as they had previously been able to do. *“Well I want to do [clean out] the cabinets; I really want to get it done*. *It all needs to be sorted out*, *but I can’t do that either*. *It makes me a bit angry with myself*.*” (F4F)*

In general, dependency first concerned the need to use assistive devices in performing the activities of daily living. This was followed by dependency on others, including informal and professional care.

Dependency on assistive devices–Some participants felt reluctant to use assistive devices (e.g., canes or rollators), because they made them feel old or disabled. In fact, some participants did not use such devices at all, even if it put them at greater risk of falling. *“That’s what I need to get over […] Then you really do feel disabled*.*” (F8F)* Most of the participants who did use assistance devices had complex care needs or were frail, although some robust participants used walking aids. Almost a third of these participants reported problems with their devices (e.g., rollator wheels getting stuck or wheelchairs that were difficult to get into an elevator). The participants saw these as reasons for not using the devices, despite their dependence on them. *“But this housing isn’t really suited to people with disabilities*. *You can’t get through the front door with a rollator*, *and they’ve got those high speed bumps at the back of the house*. *They’re so high that you can’t go there at all with your mobility scooter*.*” (C5M)*

Dependency on informal care–Most participants expressed reluctance to ask their children for help. Nevertheless, almost all of the frail participants and those with complex care needs were receiving such assistance (e.g., with domestic chores, gardening, finances) from their children, neighbors, or other informal caregivers. Most of the robust participants were not dependent on informal caregivers, although one participant had recently started asking her daughter to help with paperwork. *“Just my daughter […]*. *She often comes on a Wednesday afternoon*. *[…] If any forms need filling out*, *she does all that for me*. *[…] Because you can’t always figure it out by yourself*. *Although I’ve only just recently started doing this*. *I used to do it all myself*.*” (R10F)*

Dependency on professionals–The frail participants and those with complex care needs were often dependent on professional assistance. Those with complex care needs were particularly likely to have “care networks” consisting of both professional (domestic assistance and home healthcare) and informal care. These networks were essential to enabling these participants to continue living at home. Almost all of the frail participants were receiving at least domestic assistance. In contrast, the robust participants were not dependent on professionals, and only one was receiving domestic assistance.

The participants who were dependent on professional caregivers were generally satisfied with the quality of the services they received, despite some difficulties in arranging care and support. One participant described a situation in which she needed help but was unable to arrange it on short notice, because she had to deal with so many different people and organizations. *“It goes through so many different levels before you actually get any help […] If you need them*, *they’re not there*.*” (C2M)*

Independent living–Most participants wanted to continue living at home rather than moving into a facility, due to negative associations with loss of independence and freedom. *“Then I’ll have lost my freedom*. *I don’t want to leave here*. *I desperately want to stay here until the bitter end*.*” (C7F)* Participants compensated for their decreasing mobility by adapting their homes (e.g., installing grab bars or replacing furniture) or by wearing personal alarms. Robust participants also reported preventive strategies for maintaining independence, including staying physically active in and around the house, following a healthy diet, taking on mental challenges (e.g., puzzles and reading), and performing volunteer work. Some participants found it difficult to define what they needed to remain living at home, expressing hope that they would receive care and support if needed.

#### Decreasing social interaction

Almost all of the participants stressed the importance of social contact, although there were differences in the number and quality of social relationships. *“Look*, *we do have social contact […] it’s very*, *very important […] you can’t cope without it*. *That’s what we’ve found*.*” (C1F)*

The robust participants retained social contacts by participating in clubs, volunteering, or sharing hobbies and activities, thereby stimulating a sense of usefulness. In contrast, frail participants and, even more so, those with complex care needs, experienced changes in their relationships due to their physical impairments or illness, or due to the death of friends. *“And then someone else is gone*, *and then you have even more to cope with*. *And it hits you hard; it’s hit me hard […]*. *The companionship that was gone*. *[…] You can’t go and enjoy that person’s company any more*, *however much you would like to*.*” (F3M)* These two categories of participants also expressed a desire for more company and fun; they wanted to “get out,” (e.g. going on outings with their partners, visiting the garden center, or taking vacations).

Social interaction also differed between participants who were living alone and those who were living with partners. The latter reported less need for social contact, new or otherwise, because they still had their spouses and spent most of the day together. *“We are still able to manage*. *We like to go out together*, *we do everything together*.*” (R14M)* Participants whose partners were deceased felt a great sense of loss and found it difficult to get out to meet others.

#### Loss of control

All of the participants reported a desire to stay in control, and they considered it important to determine their own daily living schedules. Participants who received care and support from multiple and frequently changing caregivers felt a loss of control. *“I’ve seen so many faces […]*. *If you happen to be the first in line*, *then it’s early*, *but if you’re the last*, *then you’re last in line*. *It changes a lot*.*” (C6M)*

Loss of control was also reflected in the themes mentioned above. For example, one participant’s fear of becoming dependent stemmed from the assumption that dependency would lead to the loss of freedom and the ability to control what one does and when one does it. *“To be in control*, *because once you become dependent on someone else*, *your life isn’t the better for it*.*” (F3M)* Participants who became housebound because of problems with using their assistive devices (e.g., rollators, wheelchairs) experienced a profound loss of control. *“Because I can’t get away from here at all*. *I can’t get in the elevator with the rollator*. *And I can’t get back up if I go downstairs […] I’ve already managed to get the elevator really stuck [with the wheelchair]*. *My caregiver told me*, *‘Don’t do it again*.*’ It makes you nervous*. *So I’m literally a bit shut in here*.*” (C7F)*

#### Fears

Participants experienced a variety of fears related to the expected and emerging consequences of aging. These fears were intertwined throughout the aforementioned themes. Frequently mentioned fears were largely related to deteriorating health and mobility problems (e.g., fear of falling). Furthermore, some participants postponed the use of assistive devices, as they feared feeling old and disabled. Others often mentioned fears related to becoming dependent on others, with the associated fear of becoming a burden to others and losing their freedom. The interviews also revealed that all of the participants feared losing control and freedom upon moving into an institutional setting, and they therefore wanted to age in place.

### Experiences with Embrace

We found clear differences in the experiences of Embrace care and support between the participants from the three different profiles. These differences corresponded to the different care-intensity levels corresponding to the three different profiles. For the frail participants and those with complex care needs, the case manager embodied Embrace: the case managers supported, monitored, informed, and encouraged them. In contrast, robust participants reported being informed and encouraged by the Embrace group approach.

#### Relationship with the case manager

The relationships between participants and their case managers were based on equality and confidentiality; both aspects were seen as conditional for achieving productive interactions.

Equality–The participants perceived their relationships with their case managers as being based on mutual equality. Their opinions were important, and they felt in charge. *“I think she’s a friendly woman*, *and she’s on a level with you rather than looking down at you*, *and that alone is worth a lot*. *And she talks like we do [in dialect]*, *and she’s very down to earth*. *We say she’s a good one*, *and*, *as my husband says*, *we wouldn’t want to be without her*.*” (C1F)* The participants reported that their case managers took their personal preferences into account (e.g., in scheduling visits). *“Well she always asks ‘What time can I come*?*’ or ‘Does that suit you*?*’” (F4F)* In contrast, the participants reported that other healthcare professionals tended to visit when it suited their own schedules.

Confidentiality–Participants attached considerable importance to confidentiality in their relationships with their case managers, which had become even more confidential over time. They trusted that their case managers would not pass on information and that they could tell them anything. In fact, some participants were more likely to confide in their case managers than they were to confide in their own children or general practitioners. *“I don’t tell my children everything either*. *In that respect*, *I’m quite closed*. *But I’ve taken her [the case manager] into my confidence and I tell her everything*. *Then you’ve got someone you can tell it to*, *haven’t you*? *And it doesn’t go any further*.*” (C5M)*

#### Interactions

Participants perceived their interactions with Embrace professionals in several ways. They felt that they were supported, monitored, informed, and encouraged by Embrace, although the content of these interactions depended upon their profiles.

Being supported–Frail participants and those with complex care needs felt supported by their case managers. They found them highly supportive in many ways. They discussed problems with the case managers, talked about the future, and formulated plans for healthcare and other issues. Participants found it comforting that their case managers provided advice, “always knew what to do,” and were “always ready to help.” Almost all of the participants reported that they could contact their case managers if needed. *“It’s as if you’ve got some support […] I don’t want to put her [the case manager] on a pedestal*, *but she’s a real pillar of strength for us*.*” (C1F)* In addition, some participants valued the fact that their case managers also provided emotional support. They felt reassured by the words of their case managers. *“As far as empathy is concerned*, *she’s fantastic*. *And the emotional support that she gives… Her words are such a help*. *‘We’ll never*, *ever turn our backs on you*,*’ she says*.*” (C2M)* Case managers also provided social support, as their visits were enjoyable. One participant even said that it would be like “missing a friend” (C7F) if the case manager were to stop visiting her. Finally, participants received practical support from the case managers, who arranged various solutions (e.g., wheelchairs or volunteers for help with computers).

Being monitored–Frail participants and those with complex care needs were monitored by their case managers. They found it comforting that the same person visited regularly. They were able to discuss their situations with their case managers, who visited them once a month, to the participants’ satisfaction. *“Anything we tell her she brings up again the next time*. *[…] Without being prompted*, *but she’s aware of it*. *[…] And it’s the small things*, *but she takes good note of them*.*” (C1M)* In addition, participants with complex care needs found it reassuring that their case managers were in close contact with their general practitioners and that they had regular meetings. *“Yes*, *she then says ‘I’ve spoken to the doctor and he thought this or he thought that’*. *Yes*, *we’re being looked after*, *I do have that feeling*.*” (C2M)*

Being informed–Participants felt that they were being informed in various ways. The case managers played a crucial role in providing information to the frail participants and those with complex care needs. In contrast, robust participants received information on care and support options primarily during the Embrace community meetings, along with the other participants.

Participants with case managers regarded these professionals as “walking encyclopedias,” and they were able to discuss all kinds of issues with them. Most conversations tended to center on such ordinary practical matters as current health, diet, medication, care and support, assistive devices, family, and social support. *“The [case manager] is a real source of information for us*. *We regularly have questions about one thing or the other*, *and she tries to find answers for us*. *And she follows up on it too*.*” (C1F)*

The robust participants who attended the Embrace community meetings said that the information fair had provided them with useful information on care and support possibilities in their communities, as well as on clubs, volunteering, and the consequences of aging. *“You try to prevent things as much as possible*, *but I think that if something… if something were to happen to us*, *we’d know where we could get help*. *[…] A booklet containing all the information*, *I hang onto that*. *[…] I got it that morning [Embrace community meeting]*.*” (R18F)*

In addition, at the start of the intervention, the robust participants had received cards containing information on how to contact their Elderly Care Teams if necessary, although none of them could remember receiving such a card. The majority were also unaware that they could receive care and support from the Elderly Care Team, and that their general practitioners also belonged to this team.

Being encouraged–Participants received encouragement largely from their personal case managers and during the community meetings. Frail participants and those with complex care needs received suggestions from their case managers to participate in social activities (e.g. courses or the Embrace community meetings). *“For example*, *she [the case manager] brought me a leaflet*. *Because there are computer lessons for seniors here in Stadskanaal*, *‘And that’s just what you need*,*’ she said*.*” (C2M)* Some of those who attended the Embrace community meetings became inspired to participate in social activities. *“A dietician was there and [told us about] all that they do for the elderly*. *And we were all given leaflets to take home*. *They also take trips every now and then*. *[…] I went with someone I know*.*” (F4F)* The robust participants were also encouraged to engage in activities during the Embrace community meetings. For some participants, the community meetings offered a good opportunity to meet other people. *“I’d like them [Embrace community meetings] to be held more often*. *[…] Just getting to know people makes them worthwhile*.*” (R10F)*

#### Feeling in control, safe, and secure

The support, monitoring, information, and encouragement that the participants received helped them to feel in control and provided them with a feeling of safety and security. This was especially the case regarding the participants with a case manager. *“I find it a great reassurance that she [case manager] says ‘We’re here if you need us*.*” (C2M)* The participants made decisions in cooperation with their case managers, which increased their sense of being in control. In addition, participants were encouraged to participate in society, which also added to their sense of being in control. The participants also indicated that regular visits by a trustworthy case manager gave them the feeling that they were being monitored. The participants, including the robust participants, also knew what to do in case of emergency, which provided a sense of safety and security. *“If there’s anything I don’t know*, *I always talk about it with her*.*” (F12M)*

## Discussion

**Fig 2:**
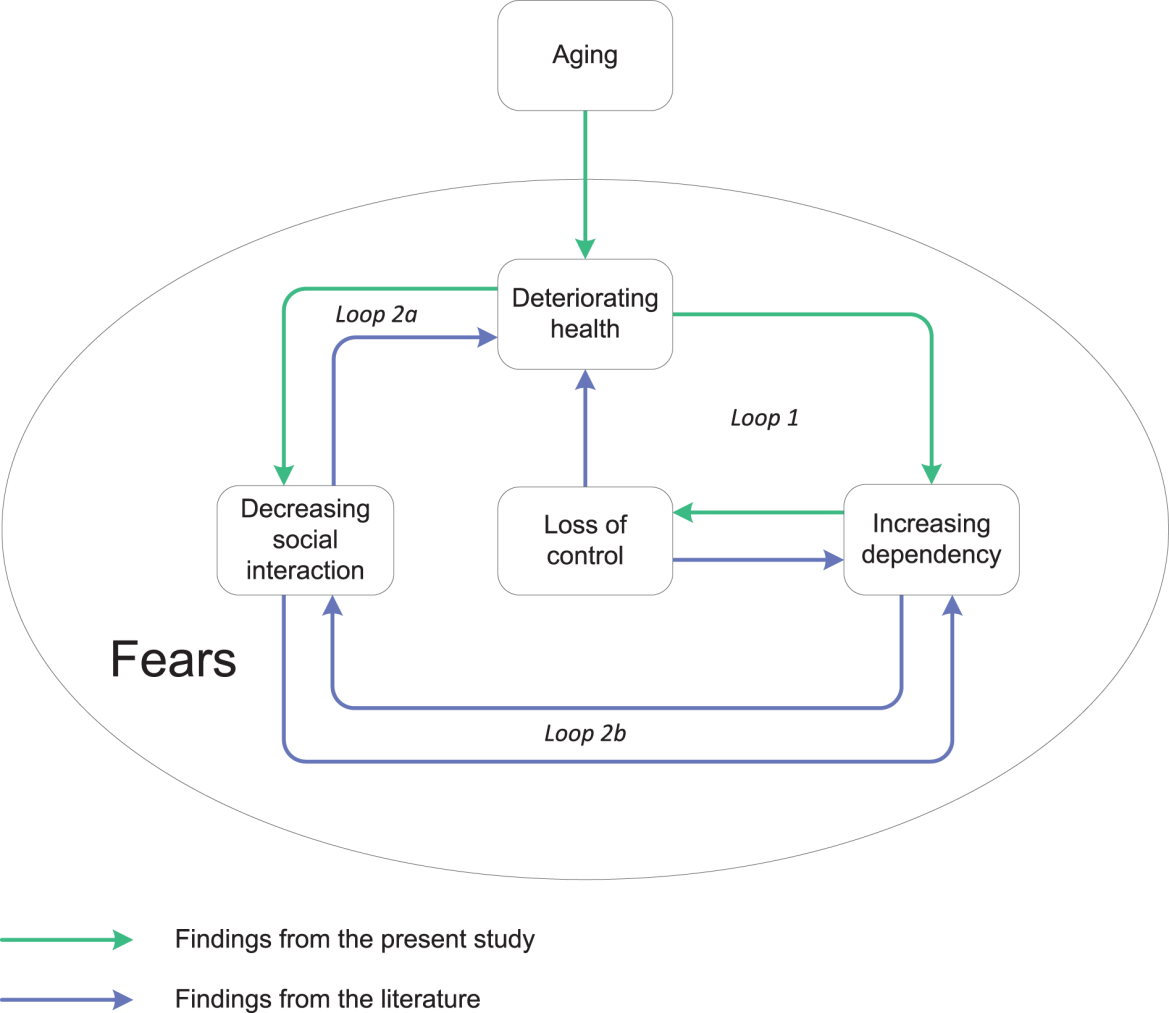
Model depicting the experiences of older adults with aging.

**Fig 3:**
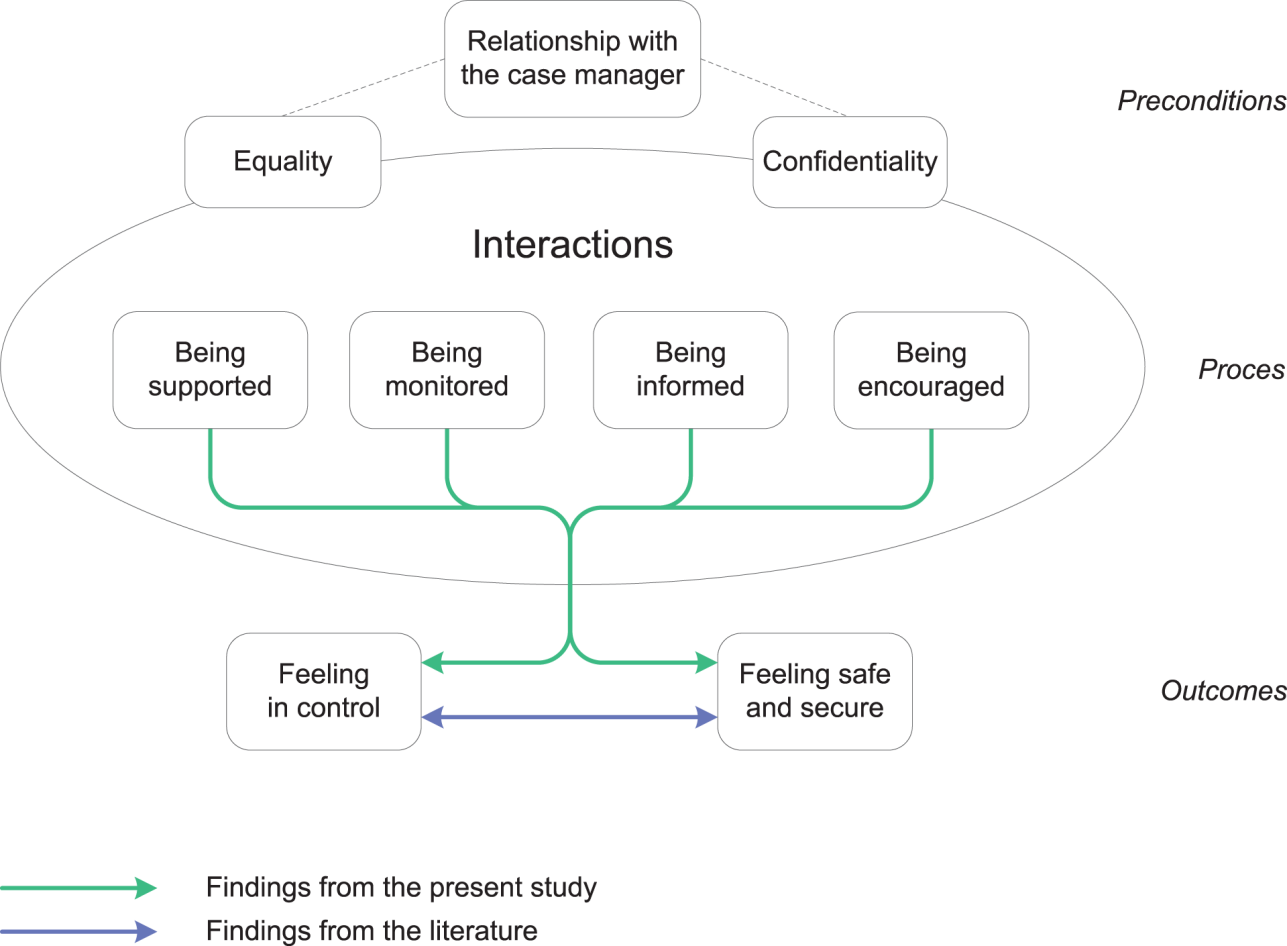
Model depicting the experiences of older adults with CCM-based integrated care and support.

This study is the first qualitative study to investigate whether an integrated care model based on the Chronic Care Model (CCM) like Embrace is indeed patient-centered and adapted to the needs of the older population. Interviews showed that participants feared increasing dependency and loss of control due to aging. The interviews also revealed that Embrace had contributed to the participant’s ability to cope with those fears and that it helped them to feel in control, safe, and secure. The results are presented in two models on 1) the experiences with aging (Fig 2) and 2) the experiences with integrated care (Fig 3). The models represent findings of our study supplemented with findings from the literature. We also compare our findings with the CCM.

### Experiences with aging

Participants reported struggling with various fears related to the consequences of aging (whether actual or expected). As their health deteriorated, their dependency increased, they became less socially integrated, and they developed a sense that they were losing control and becoming dependent on care and assistive devices. Participants who were in generally good health and were not receiving care and support expressed fears concerning these negative effects. These findings are indicated by the green arrows in the first model (Fig 2). The findings from our study are confirmed in the literature, and they can be extended by additional findings from other studies (Fig 2, purple arrows). This will be explained in the following paragraphs.

Aging can lead to several downward loops due to a deteriorating health status. The first loop that is presented in the model on consequences of aging includes the components “deteriorating health”, “increasing dependency”, and “loss of control” (Fig 2, loop 1). The negative relationship between increasing age and decreasing perceived control has been extensively documented [37, 38]. Health status can be a contributing factor [39], as confirmed in our study. According to our results, being frail–and thus being more dependent on others–reduces the sense of being in control, as previously reported by Ellefsen and colleagues [40]. In turn, this loss of control may have severe negative effects on an individual’s health condition [37, 39]. Moreover, lower perceived control may increase service usage, thus intensifying the sense of dependency [39].

Two other loops that are displayed in the first model include the component “decreasing social interaction” (Fig 2, loops 2a/b). All participants stressed the importance of social interaction, but many experienced a narrowing of their social network. For the frail participants and those with complex care needs in our study, this was largely due to their own physical limitations and illnesses or to the illness and death of friends and family. In addition, participants who were living alone–especially those who were widowed–experienced less social contact. This corroborates past research, which associates living alone with an increased risk of social isolation [41] and loneliness [42, 43] (Fig 2, loop 2a). The shrinking of social networks may have negative effects, as social support is positively related to health status and well-being [44]. Moreover, social resources and support are important pre-conditions for remaining independent of professional support [45] (Fig 2, loop 2b), which may positively influence someone’s sense of control.

One recurring issue reported by participants in relation to the consequences of aging concerned the experience of various fears. For example, almost all of the participants expressed a fear of becoming dependent on others, with the associated fear of becoming a burden to others. This is consistent with other studies [45, 46]. One major fear involved being admitted to a nursing home or home for the elderly, as this was perceived as reflecting a loss of freedom and control. This finding also mirrors results available in the literature [47, 48]. Another fear involved falling, which is known to be a common fear in older adults [49, 50]. Nevertheless, some participants were reluctant to use assistive devices, as they feared feeling old and disabled, even though such devices could reduce the risk of falling [51]. Such reluctance has been shown to be common across older adults [52].

Moreover, the smaller loops form one big loop: the downward spiral of aging. It is therefore not surprising that many Western societies–trying to reform their healthcare systems–focus on health promotion and disease prevention [10]. By implementing population-based integrated care models, like Embrace, they attempt to prevent the occurrence of these loops by providing preventive, proactive, and patient-centered care.

### Experiences with integrated care

With regard to the second research question, the interviews demonstrated that patient-centered, integrated care and support had enhanced the participants’ ability to cope with the fears associated with aging. Four types of interactions–being supported, being monitored, being informed, and being encouraged–provided participants with a sense of being in control and of being safe and secure. These findings–which are all supported by the literature–are summarized in the second model by the green arrows (Fig 3), and will be explained in the following paragraphs in relation to the CCM–as this model was the basis for the development Embrace.

The four types of interactions that we found in this study are at the level of the “productive interactions” between an “informed, activated patient” and a “prepared, proactive practice team” as described in the CCM [15]. Wagner et al. define “productive interactions” as interactions during which the team continuously monitors the status of the patient and takes action when needed (“being monitored” in our model) and supports patients to self-manage their problems (“being supported” and “being encouraged”). In addition, participants felt “being informed”, which may provide further opportunities to establish productive interactions.

Participants reported experiencing their relationships with their case managers as being characterized by equality and confidentiality. Such relationships seem to be a prerequisite for the interactions to be productive. The literature shows that trust and confidence are prerequisites for successful case management interventions. Furthermore, repeated contacts and a person-centered approach can facilitate the relationships between case managers and their clients [53, 54]. Our findings corroborate this. The strong relationships that participants experienced may in particular be due to the frequent visits of case managers and their individual-needs approach.

In contrast to the participants who had case managers, the robust participants received a low-intensity group intervention. They experienced two major types of interactions: they felt better-informed concerning options for care and support, and they felt that they were being encouraged to engage in social activities. These interactions were probably mainly due to the Self-management support and prevention program of Embrace, including community meetings and newsletters. It is an operationalization of the “Self-management support” element of the CCM. These activities are important facilitators for promoting health and maintaining the capacity for self-management [55].

The results of participation in Embrace, as experienced by participants, can be categorized into two themes: “feeling in control” and “feeling safe and secure.” These results are at the level of the “Improved outcomes” as assumed in the CCM. With regard to the first theme, participants experienced increased feelings of being in control due to interactions within Embrace. As long as people are capable of making their own decisions–as stimulated and supported in Embrace–they are able to feel in control, even while being dependent on others [56]. In addition, being well-informed can lead to an increased sense of being in control [57, 58]. Good communication and information provision can also improve patient involvement, which is one of the key concepts of patient-centered care [16].

The second theme concerns the increased feelings of safety and security. These improvements were experienced primarily by frail participants and those with complex care needs, due to the involvement of their case managers. The regular visits, the confidential relationship, and the information provided played a particularly important role in strengthening the sense of safety and security for these participants. This is consistent with earlier literature, which also demonstrates that perceived health is related to a sense of security [59–61]. The lack of a reported increase in such feelings by the robust participants is not surprising, as they were generally healthy and reported having relatively strong social networks. Although the participants did not state this explicitly, the literature suggests that sharing information on the consequences of aging and on options for care and support (as was done during the Embrace community meetings) can reduce feelings of uncertainty [61].

From the literature, it is known that “feeling in control” and “feeling safe and secure” are closely related and mutually reinforcing concepts. This is indicated by the purple arrow in the second model. Feeling in control is considered essential to feeling secure [59]. Alternatively, a sense of security can help individuals to feel that they are in control, as observed by the care recipients in the study conducted by Petersson and colleagues [60].

### The two models compared

Comparison of the two models indicates that Embrace meets the needs of older adults and that it is indeed patient-centered. Due to aging, the participants felt insecure and feared increasing dependency and loss of control. Embrace appears to have reduced these negative feelings by providing support and by monitoring, informing, and encouraging the participants. This enhanced their sense of control, safety, and security, which are important conditions for being able to age in place–the main goal of Embrace.

### Strengths and limitations

The strengths of this study include the involvement of a diverse group of participants through the application of the maximum-variation sampling technique [34]. Furthermore, opinions on Embrace, if known in advance, were not taken into account during the recruitment process, and data saturation was achieved.

One potential weakness of the study is that some older adults had difficulty reflecting on their experiences with Embrace. This might have been due to their frailty or mild memory problems or because they (like their entire generation) were not used to expressing their experiences and feelings. Nevertheless, the interviewers were able to obtain clear answers to their questions during the interviews, and the analyses of the interviews produced a clear response to the research questions.

### Implications

Additional research is needed to confirm and further develop the two models presented in this paper. For example, our findings on the effects of aging as represented in the first model should be confirmed in studies on older participants who receive other types of care. Future research should also address the possibility of self-management support and preventive interventions for the robust older population [62]. Our results support the expectation that an integrated model of elderly care can be beneficial to robust older adults. To date, however, most research on self-management support interventions has focused on patients with chronic conditions or on frail patients [63]. Further investigation is also needed with regard to the best ways to communicate with this specific older population, as most robust participants only mentioned the Embrace community meetings when talking about the support received by Embrace. They were unaware of the other care and support options offered by the Elderly Care Teams. Lastly, our findings show that this qualitative study may give a clear insight into the experiences of participants with integrated care. Therefore, future randomized controlled trials on integrated care models could similarly use qualitative research to act as a “translator” for understanding the implementation and context of the trial and may help to contextualize the findings of the trial [64].

Based on our findings, professionals should be aware of the fears that might be experienced by older adults, and they should anticipate these fears in the course of providing care and support. They should nevertheless bear in mind that some fears might not be realistic. For example, the fear of losing control by moving to an institutional setting is questionable, as the security of living in a care home might enhance the ability of older adults to feel in control (as compared to receiving care services in their own homes) [65]. In addition, professionals should realize that some clients are afraid of feeling old and handicapped. This can cause them to be reluctant to use assistive devices, even if these tools could help them live at home for longer [47] and improve their sense of security [60]. In addition, the fear of feeling handicapped can lead to social exclusion when people choose not to access resources or participate in activities [45]. Professionals should therefore focus on providing good information on aging and on explaining the advantages and disadvantages of potential interventions, as this could decrease feelings of uncertainty and concern [61].

Finally, geriatric care professionals and their managers should become aware of the importance of the role of case management in the care and support of older adults. A strong and continuous relationship between a professional and a client can provide greater insight into the client’s situation, and it is a precondition for organizing patient-centered care. Managers should therefore allow sufficient time and space for the professionals involved to invest in building strong relationships with their clients.

## Conclusion

To the best of the authors’ knowledge, this is the first qualitative study to explore the experiences of older adults with an integrated care model based on the CCM. The present study enhances existing understanding regarding what the consequences of aging mean to older adults and whether–and to what extent–their needs and wishes can be met through integrated care services such as those provided by Embrace. Our findings suggest that the prospect of becoming dependent and losing control is a key concept in the lives of older adults. The findings also demonstrate that an integrated and patient-centered model based on the CCM–like Embrace–can help participants to stay in control, even if they are dependent on others. Moreover, such a program may help participants to feel safer and more secure, in contrast to the fears of increasing dependency associated with the standard care system. Our results confirm the patient-centered character of integrated care models like Embrace, even for robust older adults.

## Supporting Information

S1 File Interview excerpts. (DOCX)

S1 Table COREQ checklist. (DOCX)
