# Supplementary material for: Experiences of Community-Living Older Adults Receiving Integrated Care Based on the Chronic Care Model: A Qualitative Study
Source: PLoS One. 2015 Oct 21;10(10):e0137803. doi: 10.1371/journal.pone.0137803 (PMC4619446; doi:10.1371/journal.pone.0137803)
Supplement: S1 File — (DOCX) [file pone.0137803.s001.docx]

**S1 File. Interview excerpts.**

I: interviewer

P: participant

PF: participant female

PM: participant male

PP: partner participant

Quotes from the manuscript are in italics and blue colored.

*……………………………………………………………………………………………………………………….*

**C1M/C1F**

*“Anything we tell her she brings up again the next time. […] Without being prompted, but she’s aware of it. […] And it’s the small things, but she takes good note of them.” (C1M)*

I: Okay, and what happens then, once she comes?

PF: Then we talk about how it’s going with with…with uhm…well, with my husband, his legs were swollen and…and….and how I’m doing. I don’t want to (unintelligible) things. (stutters) well, and then we tell her some things and, also anything special. (unintelligible) got the wheelchair going. I think it’s necessary.

[…]

I: And you had discussed this with [the case manager] first.

PF: Yes, yeah and [the case manager] said, this was going to happen, and we need to find another, but look uhm, uhm, yeah [the case manager], she, she, she’s just a normal girl, and uhm, uhm (stutters) to each his own. (unintelligible) all of that up and running.

I: Really?

PM: She knows everything that’s going on. *Anything we tell her she brings up again the next time.*

[…]

I: Okay, could you give me an example of what you talk about with her?

PM: (laughs), well no, and I forget all about it again.

I: They’re all minor things?

PF: (unintelligible) you know, and also uhm, and uhm yeah, easy things. [My husband] says it’s like a sort of pepper-upper. That’s how we see it. And well and, about [the case manager], but it’s all together, you know. That uhm, that it’s a good fit. And, and, it really is, we really are pleased with it, because there are many lonely people. [unintelligible] I said, “I can’t take care of [my son] anymore, and that makes life a bit, then life gets dull.”

I: I see, it’s different, isn’t it?

PM: [unintelligible] how’s your blood sugar, I have those stockings on, you know? And, uhm, “are your ankles still swollen?” *Without being prompted, but she’s aware of it.*

I: Yeah yeah yeah oh, then she taps your legs and says, “How’s your blood sugar?”

PM: Right, you know, right. *And it’s the small things, but she takes good note of them*. And, and if we’ve talked about something, she brings it up with the doctor. And then they talk about it, and uhm then they tell us what can and can’t be done about it. A bit, a bit of a bridge.

*……………………………………………………………………………………………………………………….*

*“I think she’s a friendly woman, and she’s on a level with you rather than looking down at you, and that alone is worth a lot. And she talks like we do [in dialect], and she’s very down to earth. We say she’s a good one, and, as my husband says, we wouldn’t want to be without her.” (C1F)*

I: Yeah yeah yeah yeah yeah yeah, and you remember when you got the, when you got that chair?

Did [the case manager] arrange that?

PF: Yes. [The case manager] is the … yeah, how can I say it? Well, I couldn’t ever do without [the case manager].

I: You wouldn’t want to do without her.

PF: No.

I: That’s nice, isn’t it?

PF: I don’t know any other way to put it. *I think she’s a friendly woman, and she’s on a level with you rather than looking down at you, and that alone is worth a lot.* *And she talks like we do [in dialect], and she’s very down to earth. We say she’s a good one, and, as my husband says, we wouldn’t want to be without her.*

I: And how often does she come?

PF: Once a month.

I: Once a month?

PF: Yes

I: Okay, and she still does?

PF: Yes.

I: Yeah, okay.

PF: And now, you know, and I can talk with her about anything uhm. No, we couldn’t put it any other way, we’re really happy with [the case manager].

PM: That’s the truth.

*……………………………………………………………………………………………………………………….*

*“It’s as if you’ve got some support […] I don’t want to put her [the case manager] on a pedestal, but she’s a real pillar of strength for us.” (C1F)*

I: And then there’s this, [the case manager] left this for you. Saying when she’ll be back, the appointment card?

PM: No, but we make an appointment, when she comes, we make an appointment for the next time

I: You’re not familiar with it, okay, and you, you do know how to reach her?

PF: Yes. We have her mobile number. And her home phone is listed in the telephone directory, and we never call her unless we need to. But when I had that heart attack, my husband called her immediately. Did call, look uhm…well and so and [the case manager] [unintelligible]. *It’s as if you’ve got some support.* Absolutely, that’s the truth. That uhm, I say maybe there’s something like that, well not for us, but for someone else, you know. That’s uhm, uhm, well uhm, now that’s helpful to you.

[…]

I: No, but I’m obviously looking for something like, you know. If we weren’t doing this, what would people be doing without?

PF: Well, now. […] No, you see, no, I just want to say uhm. *I don’t want to put her [the case manager] on a pedestal, but she’s a real pillar of strength for us.* Right, [husband]?

PM: Yes. If we have a problem, then uhm, then you really do want to talk with [the case manager].

Like that, you know. And, and, she always has an answer, or, or not, but yeah, she usually does.

*……………………………………………………………………………………………………………………….*

*“The [case manager] is a real source of information for us. We regularly have questions about one thing or the other, and she tries to find answers for us. And she follows up on it too.” (C1F)*

I: Okay, and uhm, if you think about everything that [the case manager] has been able to do in the past year. What has she done for you?

PF: I’d say a whole lot and nothing – kind of an odd thing to say, isn’t it? Yes, it could be that we just didn’t think about it, but, no, I can’t think of anything right off the bat, but *the [case manager] is a real source of information for us. We regularly have questions about one thing or the other, and she tries to find answers for us. And she follows up on it too.*

*……………………………………………………………………………………………………………………….*

*“Look, we do have social contact […] it’s very, very important […] you can’t cope without it. That’s what we’ve found.” (C1F)*

PF: That too, and that, that niece, drops by every afternoon, “Is there anything I can do for you?”

If there’s anything uhm that needs to be done, on a ladder or something, you know.

And we also had acquaintances, they’d say “I have one of those tools. I’ll leave it here.

When I leave to come here, my husband can’t climb a ladder anymore, “Is there anything else that…that….what needs to be done?” You see, it’s nice and all, but look, yeah, look [unintelligible], we’ve got so many kids. Then I tell [my husband] [unintelligible], that we’re getting even older. *Look, we do have social contact […] it’s very, very important […] you can’t cope without it. That’s what we’ve found* [unintelligible]. Look, we...

I: Then it would become really lonely, wouldn’t it?

PF: Then it gets, and so I say to [my husband], now, I can understand if people can’t read anymore, or something like that, and that they can’t do anything anymore. And then if you just sit around every day without anything to do. […]

*……………………………………………………………………………………………………………………….*

***C2M***

*“Yes, she then says ‘I’ve spoken to the doctor and he thought this or he thought that’. Yes, we’re being looked after, I do have that feeling.”*

*“It goes through so many different levels before you actually get any help […] If you need them, they’re not there.”*

I: And uhm, [the case manager] and the GP and uhm, they consult with each other.

P: Yes. [The case manager] has a good contact with [the GP].

I: Okay, and do you tell, does she tell you about it too?

P: *Yes, she then says, “I’ve spoken to the doctor and he thought this or he thought that.” Yes, we’re being looked after, I do have that feeling.*

I: And do you think that the cooperation between uhm…

P: Yes, the cooperation is good, but, yeah. I keep thinking about the time, from when we were still living on the [name street]...then we also had some kind of senior services. But then there was, on Thursday in that house, a really big house with a really big room, they always had meetings with all of those aid workers. And then they’d have a coffee break, and I’d say, “Come on. Make these people do their jobs. Make these people do something in people’s homes.” Not every – you do have to meet once a month or so – but they had these coffee parties every Thursday at the, at the government is so big, isn’t it?

I: What do you mean?

P: *It goes through so many different levels before you actually get any help.* And, and it really shouldn’t be like that.

I: And, and what kind of levels?

P: Well, the municipality, for example. First, you have to, if you need help…you have to go to the municipality first. Then a lady from the municipality comes by. And that lady consults with someone else…When it got to the point that we really needed help. Then, she was nothing (?) and I was (pretends to be short of breath), we needed help right away. The first time we got any help was four weeks later. Now, that’s when I said, “That’s just like them, isn’t it? *If you need them, they’re not there.*

I: No. And do you feel like it’s still that way? Now that you’re participating in Embrace?

P: Now I, I depend on [the case manager]. Uh, “One phone call,” she says, and you get the help you need. “Do you mean that, [case manager]?” “Yes. You can count on it.” Well, now, I really like, I really liked that about [the case manager]. I always enjoy it. Would you like a cup of coffee?

*……………………………………………………………………………………………………………………….*

*“As far as empathy is concerned, she’s fantastic. […] And the emotional support that she gives… Her words are such a help. ‘We’ll never, ever turn our backs on you,’ she says.”*

I: No, and what’s it like when, uhm, [the case manager] comes here, what uhm… Can you tell me a bit more about that?

P: Well, then…, yeah, she, she obviously asks how we’re doing. And then I can tell her that my wife is getting worse, because the end is near, that’s what our housekeeper says as well. “I’ve been working here for a year now,” she says, “And in that year, I can see that your wife has gotten worse.”

I: Yes, and what does that mean for you?

P: Well, that I’m worried about her. Yes. Because, uhm, yeah, if something were to happen to me suddenly, and I’ve said this [the case manager] too, then someone would have to come help my wife right away. Then she’d have to, yeah, she can’t remain at home. […]

I: Yes. Exactly. And, uhm, when [the case manager] is here, here uhm, is your wife present as well?

P: That’s what I said. [The case manager] usually comes on Wednesday. And then my wife is at the [daycare center], coincidentally yesterday, Tuesday. And then she said, “She’s a nice lady.” I said, “Yes. I think so too.” Yes, and [the case manager] is nothing but helpful.

I: Great.

P: And also uhm, yeah, also uhm, what oh, *As far as empathy is concerned, she’s fantastic.* But yeah, that’s just it, isn’t it? *And the emotional support that she gives… Her words are such a help. ‘We’ll never, ever turn our backs on you,’ she says.* If there’s an emergency, our neighbor will also help us to make arrangements. Because if something were to happen to me, well, she wouldn’t be able to arrange anything; my wife can’t make any arrangements. […]

*……………………………………………………………………………………………………………………….*

*“For example, she [the case manager] brought me a leaflet. Because there are computer lessons for seniors here in Stadskanaal, ‘And that’s just what you need,’ she said.”*

I: Yes. Exactly, because uhm. Could you tell me a bit more about that, what do you think of Embrace?

P: Uh well, the, the contact with [the case manager] and [case manager dementia], maybe you know them as well?

I: No, not personally.

P: Well, they’re good. [The case manager] comes once every four weeks, and my wife just happens to have gone on the bus to the daycare center. And then we have a nice chat, and then she takes care of things for me. *For example, she [the case manager] brought me a leaflet. Because there are computer lessons for seniors here in Stadskanaal, “And that’s just what you need,” she said.* A while ago, she had already sent me a volunteer. And that volunteer came one time, and then he got sick. He said, “I’ll come again when I’m better,” but he never came back. He was also, yeah, a rather odd man, but, yeah, I wish he’d been able to come back.

I: Yes, because what, what uhm, did that volunteer do for you?

P: He, he, he, he helped me with the computer.

I: Okay, yes.

P: I said, “That works out well,” because yeah, [the case manager] worked that out.

I: Great.

P: But [the case manager] thought it was a shame, because uhm, she says, “Now, there's going to be a class for seniors soon in [town], at the library. You ought to go to that.” Because I’m 88, ma’am, and yeah, I can, I can uhm do email, and I can look at my budget at the bank.

I: Now, that’s uhm

P: And I can also make payments with it, but I can’t do much else.

I: No. Now, that’s really uhm…

P: That’s a lot, but maybe I could learn a few other things. And then, if you do something, and then sometimes everything on the computer disappears, and then I don’t know how to get it back. Perhaps they could help me with that, or not.

*……………………………………………………………………………………………………………………….*

*“I find it a great reassurance that she [case manager] says ‘We’re here if you need us.”*

I: (laughs) and do you ever tell other people about Embrace? Do you ever talk about it?

P: Yes, I do. My neighbor here, and [friends] also know about it. But they had good guidance too, [my friends].

I: Because they’re participating as well?

P: Now, I don’t know if they’re participating in it, but they did have good guidance. Also from uhm... that was also a [case manager dementia]. I don’t know if you know him. He comes to see them regularly.

I: And what do you say about it? When you talk about it?

P: When I talk about it? What I say to [my friend].

I: About Embrace.

P: Now, that we still haven’t had to call on you very much. No, you come and talk, and give advice, and that’s it. And that’s fine with me. I don’t need anything else for the time being. *I find it a great reassurance that she [case manager] says “We’re here if you need us.”* That time with [the hospital] and the municipality, I had to wait for four weeks. I said, “We’ll be dead and buried by then.” And it would be a shame that my wife wouldn’t have anyone to take care of her. Because that’s the first place, you know.

*……………………………………………………………………………………………………………………….*

**F3M**

*“No, what will be, will be. […] Luckily, we don’t know what the future will bring.”*

I: If you could sum it up, what does participating in Embrace mean for you?

P: ... That I want to keep up with this, to see what’s yet to come.

I: Okay, and do you have an idea of what you might be able to expect?

P: *No, what will be, will be. […] Luckily, we don’t know what the future will bring.*

*……………………………………………………………………………………………………………………….*

*“And then someone else is gone, and then you have even more to cope with. And it hits you hard; it’s hit me hard […]. The companionship that was gone. […] You can’t go and enjoy that person’s company any more, however much you would like to.”*

I: And were things a bit worse in the past year? Has there been a change in your health?

P: Yes, particularly in the last…two years or so.

I: What’s changed? Can you describe it?

P: Yes, it was about three years ago. We were still together.

I: Okay, when was it that your wife passed away?

P: It was the second of February.

I: This year?

P: Yes.

I: Oh, it hasn’t been that long. My condolences.

P: Yes. She was also 84. We were only six weeks apart. But yes, you see, the last 18 months she was already gone. And so you just build that up, that you’re going to be taking over everything. *And then someone else is gone, and then you have even more to cope with. And it hits you hard; it’s hit me hard.*

I: That you keep having to do more?

P: Yes, that too, but also in a physical way, you know.

I: And also that you’re now living alone?

P: Uh, yes, that too, but I don’t mind being alone.

I: Really?

P: In that regard, yes. I’m fine with being alone.

I: What hit you so hard, then?

P: *The companionship that was gone.* She was like a (unintelligible, ray of sunshine?), and I was always very cheerful. People don’t believe it, but we did have a few quarrels every now and then, but we never really had a fight; that just never happened.

I: Great.

P: Yes. It was like we were two peas in a pod. Yes, exactly, exactly.

I: And the two of you lived here together?

P: Yes.

I: How long have you been living here now?

P: ...Seven years. So uhm …

I: And you say, “In the last two years, my health has been changing gradually.”

P: Yes.

I: And what has been changing exactly? You said that it was physical.

P: Yes, you could say that. Because (laughs) I’m a pretty big guy, I am. But I can no longer do anything that requires any strength or things like that. My body has thus lost all its strength.

I: Yes, exactly. That’s just happened in the past few years.

P: Right, uhm well yes, the last few years. And it’s going to keep getting worse. And you notice it. That you think when the weather is nice, then you think, “Okay, fella, you ought to be able to do it.” Then I do take my cane with me, and I might walk toward [the city], but I don’t get any farther than the crossing.

I: Yes, I see, several hundred yards.

P: Right, and then that’s it. And so what do you do? At that point, you can’t just pick yourself up and keep going. No, you just go back. *You can’t go and enjoy that person’s company any more, however much you would like to.* […]

*……………………………………………………………………………………………………………………….*

*“To be in control, because once you become dependent on someone else, your life isn’t the better for it.”*

I: If you look at your life like that, do you feel that you’re able to decide for yourself how your life is going to be?

P: ... Up to this point, yes....Yes, up to this point. If I may say so myself, I still don’t let anyone tell me which way to go.

I: Is that important to you?

P: *To be in control, because once you become dependent on someone else, your life isn’t the better for it.*

I: ... Because you’re able to manage it on your own, you have your own uhm…

P: Here you go.

I: Your own direction, as we say.

P: Right, that’s it exactly. That you’re still able to do as you please.

*……………………………………………………………………………………………………………………….*

**F4F**

*“Well I want to do [clean out] the cabinets; I really want to get it done. It all needs to be sorted out, but I can’t do that either. It makes me a bit angry with myself.”*

I: And you just now said that you, that you’re not so happy because…

P: *Well I want to do [clean out] the cabinets; I really want to get it done. It all needs to be sorted out, but I can’t do that either. It makes me a bit angry with myself.*

I: (laughs)

P: But yeah, that doesn’t help either. Well, it’ll be okay eventually. I have different medications again, and I’ve not them for very long, so I think I’ll make it.

I: And are there other things that you wish you could do? Or that you’d like to change?

P: … Well, yes, change. Now, sometimes I, like I sometimes used to have, I had various things to look forward to. Going there, and there, and now I sometimes think, why don’t I just stay right here at home. But then I do go, I think, because you do have to get out once in a while. If that’s due to getting old, I don’t know. I’ve noticed it about myself.

*……………………………………………………………………………………………………………………….*

*“A dietician was there and [told us about] all that they do for the elderly. And we were all given leaflets to take home. They also take trips every now and then. […] I went with someone I know.”*

I: Yes, that’s absolutely fine...And if I’m not mistaken, you have one of [the case managers’] appointment cards, is that right? A contact card.

P: Yes, I have a card with her telephone number, and if there’s a problem or anything, I can just call her. But I’ve never had to call her, so that’s worked out well so far.

I: Yes, and would you do it if you had to? Would you call her?

P: Now, I think so. Yes, I think I would. We recently went to one of those [?] places. She gave me a note, you know, “You could go too.” About everything as well uhm, *A dietician was there and [told us about] all that they do for the elderly. And we were all given leaflets to take home. They also take trips every now and then* with a van, they go places. And that’s supposed to be really nice. Now, I didn’t know anything about that, *I went with someone I know.*

I: And how was it?

P: Now, it was quite enjoyable. Yes, and you pick up new ideas.

[…]

I: And those brochures, did [the case manager] give them to you?

P: No, we got them there. There were all of these booths, you know. You could even have a bite to eat, but I didn’t do that.

I: And how did you know about that?

P: Well, [the case manager] gave me a note that said it was there. And then, then I ran into her there, and she said, “Well, golly, you’re here too,” and I said, “Indeed,” and I thought, “It must really be important.”

I: Yes, that’s nice. And did you go there alone, or did you go together?

P: No, with someone I know.

*……………………………………………………………………………………………………………………….*

*“Well she always asks ‘What time can I come?’ or ‘Does that suit you?’”*

I: That’s enough. Good. And the time that she [the case manager] is here, do you think it’s long enough?

P: *Well she always asks “What time can I come?” or “Does that suit you?”*

I: Okay. And how long is she here?

P: About an hour, I guess, and sometimes a bit longer.

PP: Never more than an hour, right.

P: It depends on what we need to talk about.

I: Yes, and before you were participating in Embrace, did anyone ever come by?

P: No, not at all.

I: So you’d say this is really something new?

P: Yes, yes it is.

I: And what do you think of the change? …that she comes here now?

P: Well, yeah, like I said, you can get things off your chest, you know.

*……………………………………………………………………………………………………………………….*

**C5M**

*“But this housing isn’t really suited to people with disabilities. You can’t get through the front door with a rollator, and they’ve got those high speed bumps at the back of the house. They’re so high that you can’t go there at all with your mobility scooter.”*

I: And do you feel a bit safe, here in your home?

P: Certainly. I’m not afraid here at all…There’s always the chance that something, that there could be a fire or something like that. *But this housing isn’t really suited to people with disabilities. You can’t get through the front door with a rollator, and they’ve got those high speed bumps at the back of the house. They’re so high that you can’t go there at all with your mobility scooter.*

I: Where do you keep it then, the mobility scooter?

P: In the garage.

I: In back?

P: Yes.

I: Okay, so the house is not really very well adapted for…

*……………………………………………………………………………………………………………………….*

*“I don’t tell my children everything either. In that respect, I’m quite closed. But I’ve taken her [the case manager] into my confidence and I tell her everything. Then you’ve got someone you can tell it to, haven’t you? And it doesn’t go any further.”*

I: And what do you see as the difference between the time before [the case manager] came and now that she is coming?

P: Well, just a normal conversation for a change. Like I said, I tell her everything, she knows everything, at least everything about me, you could say.

I: Okay. How does that make you feel?

P: Yeah, I feel…how can I say it? Yeah, I like it.

I: Is it also a relief?

P: Yes, yes it is. You have one person who you can tell everything, and you really need that. And you could tell it to your children too. The children also know every...no, not everything. They know my, I was just talking about my PIN, the children don’t yet know my PIN either.

I: Good for you (laughs). You have to keep some things to yourself.

P: *I don’t tell my children everything either. In that respect, I’m quite closed.*

I: “You’re quite closed.” Yes. I think that it also has to do with age, you know, with the generation.

P: Yes, I think so too. *But I’ve taken her [the case manager] into my confidence and I tell her everything. Then you’ve got someone you can tell it to, haven’t you? And it doesn’t go any further.* (clock strikes)

*……………………………………………………………………………………………………………………….*

**C6M**

*“I’ve seen so many faces […]. If you happen to be the first in line, then it’s early, but if you’re the last, then you’re last in line. It changes a lot.”*

I: […] and now what I actually wanted to know first was what a normal day looks like for you.

P: What it looks like?

I: Yes. What you do on a…?

P: Now, I’ll tell you. It starts in the morning, you know. And it can change a lot; that’s just how it is, it’s related to that. Every day, the nurses come by. In the morning, they get her dressed, bathe her. And that’s how it starts, and the nicest thing is [unintelligible], as you can see, I can’t walk very well anymore. And I’ve got other problems as well, but I usually get up. I get up, and she stays in bed…There is a time difference. At first, they were at the door at 8:00 AM. That was a bit early for me. […]

[…]

I: Okay. Do they usually come every morning?

P: Every morning. They’re always there.

I: And that, that’s the nurses…

P: But the time varies a lot, you know. They’re given a route… […]

[…]

P: Well, I think it’s fantastic. They come here, but it’s still, this is all going to be on that, isn’t it? [points to the recording device] But sometimes a man comes by as well, you know, and he’s supposed to bathe her down there as well… and that, that uh…And my wife doesn’t like that very much.

I: No, and you don’t have any...you can’t say anything about that?

P: No, because there are a lot of them. We’ve been working here now almost since August, so almost three months. *I’ve seen so many faces,* so many people come here, and each of them is assigned a route. *If you happen to be the first in line, then it’s early, but if you’re the last, then you’re last in line. It changes a lot.* So…

I: It changes a lot,

P: It changes a whole lot.

*……………………………………………………………………………………………………………………….*

**C7F**

*“Because I can’t get away from here at all. I can’t get in the elevator with the rollator. And I can’t get back up if I go downstairs […] I’ve already managed to get the elevator really stuck [with the wheelchair]. My caregiver told me, ‘Don’t do it again.’ It makes you nervous. So I’m literally a bit shut in here.”*

I: Okay. Do people come here?

P: Certainly. Yes. *Because I can’t get away from here at all. I can’t get in the elevator with the rollator. And I can’t get back up if I go downstairs.* I can do like this in the wheelchair, but the elevator is almost never level. And then there’s so much difference, sometimes even more than that, and then I can’t get it over the edge.

I: Then you can’t manage by yourself?

P: No.

I: You need help.

P: Yes. Then there has to be someone around. *I’ve already managed to get the elevator really stuck [with the wheelchair]. My caregiver told me, “Don’t do it again.” It makes you nervous.* *So I’m literally a bit shut in here.* Yes, the fact that someone comes to go with me...well, of course, my children take me out for a bit. A few times, when the weather’s been really nice, [the case manager] has taken me for a walk.

I: Oh, that’s nice. Oh, that’s great. Yes.

P: “You have to get out for a bit, you know. Shall I put your jacket on?” I said...“Do you have time for that?” “Okay, then we can talk on the way.” And that’s what we did.

*……………………………………………………………………………………………………………………….*

*“Then I’ll have lost my freedom. I don’t want to leave here. I desperately want to stay here until the bitter end.”*

I: ... And how important is it for you to be able to give a bit of your own structure to your life?

P: Very important... [unintelligible] it’s such a nice place for me and eh...what do you call that place?

I: [Name assisted living complex 1].

P: What?

I: [Name assisted living complex1]?

P: No, [name assisted living complex 2].

I: Oh, [name assisted living complex 2], okay.

P: “The rooms there were really nice, you look out on the street, lovely.” I said, “Now, that’s really nice.” “It’s not going to work out, is it?” she said. I said, “No.”

I: And why not?

P: I said, “You can find another sweet lady for it.”

I: …and what led you to say that?

P: *Then I’ll have lost my freedom. I don’t want to leave here. I desperately want to stay here until the bitter end.*

I: I can imagine. After all, it’s your house.

P: Well, yes it is, but no, it’s not a nice house anymore.

I: ... But it’s home, for now.

P: Yes.

*……………………………………………………………………………………………………………………….*

*One participant even said that it would be like “missing a friend” (C7F) if the case manager were to stop visiting her.*

I: Suppose that the project were to stop. What would you miss if [the case manager] stopped coming?

P: Right, then I’d have lost a *friend* (laughs).

I: Would you regret that?

P: Yes…I think that she’d still come by again anyway.

I: Okay. That she’d just pay a personal visit.

P: Yes. I think that. I can’t know for sure. I might be wrong [unintelligible]. Do you think that’s the way it would be?

*……………………………………………………………………………………………………………………….*

**F8F**

*“‘That’s what I need to get over […] Then you really do feel disabled.”*

I: Yes. That’s right. And has [the case manager] been able to do anything else for you?

P: Now, like I said before, she does...once I was having trouble with the rollator, she had uhm requested it from the doctor’s assistant and... […] Now, I finally got it, but I had to go through a real hassle with the Silver Cross. [The case manager] also wrote a letter. I recently wrote a long letter, explaining everything, and then they finally…paid back 79 euros.

I: And [the case manager] had called about that as well?

P: Yes. Yes, after I had already written to them. And that took months, even though everyone here had been reimbursed for their rollators immediately. I put that in the letter too. And uhm…they finally got around to doing it... It was 95 euros. That thing is standing in the hallway. I’ve never even used it (laughs).

I: And why is that? (laughs)

P: Well, it’s hard for me to use it right now.

I: And how…

P: *That’s what I need to get over.*

I: What do you need to get over?

P: Yeah. I don’t know, but it...yeah *Then you really do feel disabled,* I’ll just put it that way. And that’s the point. Because if the weather’s nice, I’d be able to walk to the park. That would be really nice. But I just have to make myself do it. Then you really do feel disabled.

I: …Do you need someone to go with you?…

P: Yes. You actually need to be pushed into doing it.

I: Right (laughs)

P: It’s true. You know, you can only rely on yourself. I’ve been alone for 27 years and so...solving all your problems on your own. You do have four children, but you can’t go to them for everything. You just don’t do that.

*……………………………………………………………………………………………………………………….*

**F9F**

*“I feel… I’m generally more anxious, particularly in the dark and when driving, that sort of thing.”*

I: Okay, fine. How safe do you feel? You were just talking about burglary.

P: ... When we had this house built 20 years ago, we had an alarm system set up. We actually never used it, but someone broke in last year... They tried to, but at...this door, the door to the utility room, the door next to the side room, because you can see, they had installed a device. They didn’t succeed, and from that time on, we’ve been turning on the alarm system every evening.

I: Yes.

P: So I feel safe now.

I: Now, yes. And you look to see who’s at the door, that they obviously also…

P: Yes. When the doorbell rings, when it gets dark, now, I actually look through the little window all the time. […] But it’s not just because of the burglary here, but in general, you know? I mean, when I’m leaving the [hotel] on Monday evening, after playing bridge, and it’s 11:30 PM, and it’s dark and quiet...I have to say that I’m becoming more afraid. *I feel… I’m generally more anxious, particularly in the dark and when driving, that sort of thing.*

I: And why is that?

P: Yeah, I don’t really know where that’s coming from. Well, I have talked about it a few times with my husband. We’ve been really frightened by the television, with everything that’s going on around the world. Not just in the Netherlands, but around the world. What those young people are doing, it’s gotten incredibly empty here, you know, and that whole mess in Haren, you know. That’s frightening. It makes you afraid, and it’s all broadcast on television and that…yeah, you have to process all of that.

I: That brings it into your own life.

P: Yes...and you can’t run like you could in the past.

*……………………………………………………………………………………………………………………….*

**R10F**

*“That you’re not dependent on someone else […] because you see it here from close up: someone arrives in the morning to wash you, at lunch time to make you a hot meal and wash up again, and then in the evening to get you ready for bed. I hate the idea of that.”*

I: Yes, exactly... And do you feel as if you can structure your own day?

P: Come again?

I: Do you feel as if you can decide for yourself what you are going to do during the day?

P: Oh, yes.

I: Yes.

P: It’s all up to me.

I: And how important is that to you?

P: Very important. *That you’re not dependent on someone else* that makes a big difference. And … *because you see it here from close up: someone arrives in the morning to wash you, at lunch time to make you a hot meal and wash up again, and then in the evening to get you ready for bed. I hate the idea of that.* Yes, indeed.

*……………………………………………………………………………………………………………………….*

*“Just my daughter […]. She often comes on a Wednesday afternoon. […] If any forms need filling out, she does all that for me. […] Because you can’t always figure it out by yourself. Although I’ve only just recently started doing this. I used to do it all myself.”*

I: And do you have any other assistance from family or friends?

P: No. Every man for himself.

I: “Every man for himself,” right.

P: Now, if I were...if I couldn’t manage, I could...call one of my children, and they’d come, you know? But, yeah, you don’t do that right away.

I: No, and why wouldn’t you do that right away?

P: No, oh, I don’t know. If you can manage on your own, you should do it yourself.

[…]

P: *Just my daughter.* She doesn’t come on Sunday, but *she often comes on a Wednesday afternoon.* Then she comes right after getting off from work…then she drops by. *If any forms need filling out, she does all that for me.*

I: Okay, so she does help you with some paperwork, uhm…?

P: Yes, yes, yes. *Because you can’t always figure it out by yourself.*

I: No, exactly.

P: *Although I’ve only just recently started doing this. I used to do it all myself.* Well, we were always in business and so uhm...you know, then you know what to do. I: So you know what to do, right. My whole life (laughs), yes.

I: And why, why is it more difficult for you now?

P: Well, I’m not able to get things uhm...well organized anymore.

I: No.

P: I could manage. But I can’t handle it as quickly as I used to.

I: No, exactly.

P: But yeah, what do expect when you’re 88?

*……………………………………………………………………………………………………………………….*

*“I’d like them [Embrace community meetings] to be held more often. […] Just getting to know people makes them worthwhile.”*

I: Yes, exactly (laughs). Yes, as long as it’s just every now and then uhm...you know, right? … And uhm...let’s see, we’ve now discussed a whole lot of things. Now just a general question about Embrace, can you think of anything that you think should be improved or done differently, or is there anything else you’d like to see happen...?

P: No, absolutely nothing. I don’t make any demands anymore. I think that, that, uh, you people will decide or... [unintelligible] do it yourselves.

I: (laughs) Yes, exactly.

P: Yes. Am I right? (laughs)

I: Yes (laughs). Yes, that’s true.

P: Like I said, *I’d like them [Embrace community meetings] to be held more often*

I: Yes, exactly.

P: *Just getting to know people makes them worthwhile.*

I: Okay. Are you referring to new contacts or also to meeting people you already know there?

P: No, just…just contacts, just being together. Whether you know them or not. Now, I’m the sort of person who doesn’t tend to hold back, even when I don’t know anyone. But look, that type of contact is nice.

I: Yes, exactly. And would you, for example, if they were to be held more frequently in another town, would you go there as well?

P: Well, that depends, you know, how you’re going to get there.

I: Yes, exactly.

P: I’d prefer it to just be here in the [community center] (laughs).

*……………………………………………………………………………………………………………………….*

**F12M**

*“If there’s anything I don’t know, I always talk about it with her.”*

I: This is the appointment card, yes, and what do you discuss with [the case manager]?

P: A little bit about everything.

I: Can you give an example?

P: ... It’s really easy for me to talk to her. I’ll leave this here for now.

I: We’ll clear that away in a little while.

P: Yes, very good...it’s really easy for me to talk with that woman, yes.

I: About your health, or...?

P: Yes. Yes, about health, and everything. And the way in which I interact with people. Now, they’re really pleased with that. I get along with everyone.

[…]

I: And if you need anything, do you discuss it with [the case manager] as well?

P: Yes. *If there’s anything I don’t know, I always talk about it with her.*

*……………………………………………………………………………………………………………………….*

**R14M/R14F**

*“We are still able to manage. We like to go out together, we do everything together.” (R14M)*

I: Yes. Okay. And…now that you’re participating in Embrace, do you feel that things have changed for you?

PF: No.

PM: No, no, because we really don’t need to change anything for the time being. *We are still able to manage. We like to go out together, we do everything together.* It’s not as if…my wife says, “Listen here, I don’t like yard work, I’ll do the housekeeping.” I don’t say, “…I don’t like housekeeping, I’ll do the yard.” We do everything together. And some people seem to have noticed that we do everything together. When we go somewhere, we hear, “Oh, you know just as much about it as your husband.”

*……………………………………………………………………………………………………………………….*

**C15F**

*“But to say ‘I feel fit,’ no, I won’t ever be able to say that again.”*

I: Yes. That’s good... How would you describe your health? …Or how do you feel?

P: Well…

I: Difficult question...

P: Yes. It’s a difficult question, and it changes a lot. Sometimes, I have periods when I’m very dizzy...it’s almost impossible to be up and about. Other time, I’m very nauseous...and then exhausted, it’s always something. You’re never in as good a shape as you were in the past. Everything’s going well, and the flushing is going well, and the blood values are relatively good and, if not, we’ll change to another fluid. *But to say ‘I feel fit,’ no, I won’t ever be able to say that again.*

*……………………………………………………………………………………………………………………….*

*“You become so dependent if you require assistance with everything.”*

I: Because…because you feel safe at home, with regard to falling or…?

P: Now, not always. I’m working on that with the physical therapist, yes. On one of those wiggle boards, you know.

I: Oh, yes (laughs).

P: …And then I have to do exercises…on the table for the leg muscles, to strengthen those muscles a bit. Yes. And then you try to...to stay in the running, you know?

I: Yes, exactly. And that makes you feel safe at home? Because you do some…

P: When I’m okay, yes. But when I’m dizzy, then…no, then I sometimes have problems, you see.

I: Yes…and what do you do about it?

P: Well, then my husband goes down the stairs ahead of me, and I put my hands on his shoulders, and we get by like that. But it’s getting...yeah, right now everything’s going okay again, luckily, but sometimes, you just can’t manage.

I: No, and how important is it to you, for you to feel safe?

P: Well, very important, see... *You become so dependent if you require assistance with everything.*

*……………………………………………………………………………………………………………………….*

**R18F**

*“You try to prevent things as much as possible, but I think that if something… if something were to happen to us, we’d know where we could get help. […] A booklet containing all the information, I hang onto that. […] I got it that morning [Embrace community meeting].”*

I: No, no. I understand. And there was also a short questionnaire about if you…for example, if anything had changed in your health situation or living situation, when you could contact Embrace…do you have that? Do you still remember it?

P: No, I know... Yes, as long as, look…if something were to happen to us, yes, you can obviously never know…

I: No, exactly.

P: Because now with the snow, I’m always really cautious about... going outdoors, and also about walking. I do that all the time…I don’t want to fall. Last year, I feel off my bike, but…I’m not going to let that happen again. If it’s snowing or sleeting, I just don’t ride my bike anymore...I don’t do that anymore. But…*You try to prevent things as much as possible, but I think that if something… if something were to happen to us, we’d know where we could get help.* I know that.

I: Yes, because…because anyone…

P: … and you would obviously be treated by a doctor and such as well, I think.

I: Yes. Exactly.

P: Now, perhaps you might have to know certain organizations, but they gave us that...*a booklet containing all the information, I hang onto that.* Uh..

I: Yes.

P: Yes, you can never know how your life will end, but yes...you have to just leave that...leave that to fate.

I: Yes. And with the booklet, you mean…a municipal service guide or something like that?

P: Yes, now, it’s not…but *I got it that morning [Embrace community meeting]* and it contains a variety of things. Yes, and I keep that together, in case I ever need it... [unintelligible]
