# Supplementary material for: Experiences of Community-Living Older Adults Receiving Integrated Care Based on the Chronic Care Model: A Qualitative Study
Source: PLoS One. 2015 Oct 21;10(10):e0137803. doi: 10.1371/journal.pone.0137803 (PMC4619446; doi:10.1371/journal.pone.0137803)
Supplement: S1 Table — (DOCX) [file pone.0137803.s002.docx]

**S1 Table.** **COREQ checklist.**

| **No** | **Item** | **Guide questions/description** | **Answer** |
| --- | --- | --- | --- |
| **Domain 1: Research team and reflexivity** | | | |
| **Personal Characteristics** | | | |
| 1. | Interviewer/facilitator | Which authors conducted the interview or focus group? | ASF and KS |
| 2. | Credentials | What were the researcher's credentials? *E.g. PhD, MD* | ASF – PhD  KS – MSc |
| 3. | Occupation | What was their occupation at the time of the study? | ASF – Researcher  KS – Researcher and communication advisor |
| 4. | Gender | Was the researcher male or female? | ASF and KS are both female |
| 5. | Experience and training | What experience or training did the researcher have? | ASF – has previous qualitative health research experience and training in qualitative research  KS – has experience with applied qualitative health research and training in qualitative research |
| **Relationship with participants** | | | |
| 6. | Relationship established | Was a relationship established prior to study commencement? | No |
| 7. | Participant knowledge of the interviewer | What did the participants know about the researcher? e*.g. personal goals, reasons for doing the research* | Interviewers introduced themselves to the participants and provided information on the background and aim of the study |
| 8. | Interviewer characteristics | What characteristics were reported about the interviewer/facilitator? e.g. *Bias, assumptions, reasons and interests in the research topic* | The interviewers provided information on the purpose of the interview to the participants prior to the interview. |
| **Domain 2: study design** | | | |
| **Theoretical framework** | | | |
| 9. | Methodological orientation and Theory | What methodological orientation was stated to underpin the study? *e.g. grounded theory, discourse analysis, ethnography, phenomenology, content analysis* | Grounded theory |
| **Participant selection** | | | |
| 10. | Sampling | How were participants selected? *e.g. purposive, convenience, consecutive, snowball* | Maximum-variation sampling was applied. |
| 11. | Method of approach | How were participants approached? e*.g. face-to-face, telephone, mail, email* | Frail participants and participants with complex care needs were selected and invited by telephone by their case manager. Robust participants were recruited face-to-face by project managers of Embrace during the Embrace community meeting. |
| 12. | Sample size | How many participants were in the study? | 23 |
| 13. | Non-participation | How many people refused to participate or dropped out? Reasons? | None |
| **Setting** | | | |
| 14. | Setting of data collection | Where was the data collected? e*.g. home, clinic, workplace* | At home |
| 15. | Presence of non-participants | Was anyone else present besides the participants and researchers? | Only once: a spouse who was not participating in Embrace. |
| 16. | Description of sample | What are the important characteristics of the sample? *e.g. demographic data, date* | Gender, age, Embrace profile, living situation, degree of urbanization, educational level |
| **Data collection** | | | |
| 17. | Interview guide | Were questions, prompts, guides provided by the authors? Was it pilot tested? | Interviews were conducted following a semi-structured interview guide. Two pilot interviews were performed. |
| 18. | Repeat interviews | Were repeat interviews carried out? If yes, how many? | No |
| 19. | Audio/visual recording | Did the research use audio or visual recording to collect the data? | Interviews were audio-recorded. |
| 20. | Field notes | Were field notes made during and/or after the interview or focus group? | Notes were made at the time of the interview to keep track of the topics discussed. |
| 21. | Duration | What was the duration of the interviews or focus group? | Approximately 60-90 minutes per interview. |
| 22. | Data saturation | Was data saturation discussed? | Yes |
| 23. | Transcripts returned | Were transcripts returned to participants for comment and/or correction? | No |
| **Domain 3: analysis and findings** | | | |
| **Data analysis** | | | |
| 24. | Number of data coders | How many data coders coded the data? | Three |
| 25. | Description of the coding tree | Did authors provide a description of the coding tree? | No |
| 26. | Derivation of themes | Were themes identified in advance or derived from the data? | Themes were derived from the data. |
| 27. | Software | What software, if applicable, was used to manage the data? | Kwalitan 6.0 software |
| 28. | Participant checking | Did participants provide feedback on the findings? | No |
| **Reporting** | | | |
| 29. | Quotations presented | Were participant quotations presented to illustrate the themes/findings? Was each quotation identified? e*.g. participant number* | Quotations are provided and identified with a code referring to the first letter of the profile; the number of the interview and gender. |
| 30. | Data and findings consistent | Was there consistency between the data presented and the findings? | Yes |
| 31. | Clarity of major themes | Were major themes clearly presented in the findings? | Yes |
| 32. | Clarity of minor themes | Is there a description of diverse cases or discussion of minor themes? | Yes |
